# Integration of metabolic databases for the reconstruction of genome-scale metabolic networks

**DOI:** 10.1186/1752-0509-4-114

**Published:** 2010-08-16

**Authors:** Karin Radrich, Yoshimasa Tsuruoka, Paul Dobson, Albert Gevorgyan, Neil Swainston, Gino Baart, Jean-Marc Schwartz

**Affiliations:** 1 Faculty of Life Sciences, University of Manchester, Manchester M13 9PT, UK; 2 Helmholtz-Zentrum München, Technische Universität München, 80333 München, Germany; 3 National Centre for Text Mining, University of Manchester, Manchester M1 7DN, UK; 4 Japan Advanced Institute of Science and Technology, Nomi, Ishikawa 923-1292, Japan; 5 School of Chemistry, University of Manchester, Manchester M1 7DN, UK; 6 Cell Systems Modelling Group, School of Life Sciences, Oxford Brookes University, Oxford OX3 0BP, UK; 7 Faculty of Health and Medical Sciences, University of Surrey, Guildford, Surrey GU2 7XH, UK; 8 Manchester Centre for Integrative Systems Biology, University of Manchester, Manchester M1 7DN, UK; 9 VIB Department of Plant Systems Biology/Department of Biology, Ghent University, 9052 Gent, Belgium

## Abstract

**Background:**

Genome-scale metabolic reconstructions have been recognised as a valuable tool for a variety of applications ranging from metabolic engineering to evolutionary studies. However, the reconstruction of such networks remains an arduous process requiring a high level of human intervention. This process is further complicated by occurrences of missing or conflicting information and the absence of common annotation standards between different data sources.

**Results:**

In this article, we report a semi-automated methodology aimed at streamlining the process of metabolic network reconstruction by enabling the integration of different genome-wide databases of metabolic reactions. We present results obtained by applying this methodology to the metabolic network of the plant *Arabidopsis thaliana*. A systematic comparison of compounds and reactions between two genome-wide databases allowed us to obtain a high-quality core consensus reconstruction, which was validated for stoichiometric consistency. A lower level of consensus led to a larger reconstruction, which has a lower quality standard but provides a baseline for further manual curation.

**Conclusion:**

This semi-automated methodology may be applied to other organisms and help to streamline the process of genome-scale network reconstruction in order to accelerate the transfer of such models to applications.

## Background

Metabolism is perhaps the best characterised of all molecular interaction networks in biology. Large amounts of data relating to metabolic reactions are available to date, but despite this wealth of information metabolic phenotypes remain difficult to predict accurately [1]. The reconstruction of the genome-scale metabolic network of an organism represents a major milestone toward better understanding of its properties. While metabolic pathways are convenient abstractions to represent routes of biochemical conversions of small molecules in an organism, their definition is often arbitrary and varies between sources [2]. The pathway paradigm fails to provide an integrated view of interactions, regulatory and control mechanisms, which act across and with no regard of pathway boundaries. For that reason, a growing number of genome-scale metabolic networks have been constructed in recent years, most notably for microorganisms [3-7] but also for animals and humans [8-10]. The applications of such reconstructions are plentiful, encompassing metabolic engineering studies to design strains overproducing desired products, the prediction of genes responsible for orphan reactions, the determination of active reactions for a given environmental condition, the identification of coupled reaction sets, and evolutionary studies [11]. Genome-scale metabolic reconstructions were also used to predict potential new antibiotic targets [12].

However, the process of reconstructing the genome-scale metabolic network of an organism remains very labour-intensive. It has been observed that the number of available metabolic models is smaller than 1% of that of fully sequenced genomes [13]. There is therefore a clear need to accelerate and streamline the process of network reconstruction. Fully automated reconstructions on the other hand remain imprecise, mainly because of inherent limitations and errors contained in individual databases [14]. Common sources of problems are the non-uniqueness of metabolite identifiers (some compounds being represented by generic classes such as "alcohol"), unbalanced atomic species arising from an incorrect stoichiometry or formula for one or more reactants, incorrect or missing cofactors, and enzymes catalysing more than one reaction [15]. Additional problems are caused by the lack of usage of standards for the annotation of metabolites and reactions, making the comparison of different models extremely difficult.

In this work, we present an original semi-automated methodology aimed at accelerating the process of metabolic network reconstruction, while at the same time avoiding the loss of accuracy consecutive to a fully automated reconstruction. The underlying principle of our approach is to confront and integrate different sources of data. Different databases may contain errors or gaps about enzymes, reactions and metabolites present in an organism, but if data sources are independent, the likelihood that the same error would appear twice would be expected to be of an order of magnitude smaller than the frequency of errors in each data source. By creating an intersection between two databases, differences can be identified and assessed for further curation. The resulting process can be defined as semi-automatic because the processes of extraction and comparison between databases can be largely automated, yet a manual curation remains necessary to analyse and solve discrepancies.

As an application of our methodology, we present results of the integration of metabolite and reaction data for the model plant *Arabidopsis thaliana*. Plants offer a wide range of potential applications for metabolic modelling and engineering, which include the generation of pharmaceutical compounds, increasing the production of key secondary metabolites of commercial interest, improving yield and nutritional quality of crops. Despite such promising applications, the challenges of metabolic reconstruction are compounded in plants and only recently were the first genome-scale metabolic models of *Arabidopsis *reported [16,17], which were constructed by manual curation. The reconstructions presented in this study were developed independently through a semi-automated process integrating data from two sources, namely AraCyc [18] and Kegg [19].

## Methods

In order to map metabolites between Kegg and AraCyc, several features of the data were taken into account. Two compounds were defined as being identical only if all features were positively matched. These features included compound names, chemical formulae and structures, and enzymes catalysing reactions where the considered metabolites participate.

### Compound name similarity

Names of metabolites may differ between databases for several reasons. Many chemical compounds are commonly known under multiple names, and all of their names are not necessarily indicated in all databases. Furthermore, there is no universal and common identifier between Kegg and AraCyc, as compounds are sometimes referred by their ChEBI (Chemical Entities of Biological Interest [20]), CAS (Chemical Abstracts Service [21]) or PubChem [22] identifier. For example, there are five different names listed for the Kegg entry C00022 (pyruvate, pyruvic acid, 2-oxopropanoate, 2-oxopropanoic acid, pyroracemic acid), and nine different possibilities for the same compound in AraCyc (pyruvate, BTS, alpha-ketopropionic acid, acetylformic acid, pyroracemic acid, 2-oxopropanoic acid, pyruvic acid, 2-oxopropanoate, 2-oxo-propionic acid). If at least one of these entries is the same in both databases, the identification of matching metabolites is straightforward. However there are many cases where no perfect match can be found. For example the Kegg compound C10434 is named 5-O-Caffeoylshikimic acid, and the same compound in AraCyc has the name caffeoylshikimate.

For this reason a string similarity algorithm, originally developed for the identification of gene/protein name similarity, has been employed to compare metabolite names [23]. This algorithm uses logistic regression to compute the similarity between strings by incorporating a variety of features. A training data set has to be supplied in order to teach the program which differences can be treated as similar and which ones are not allowed. It is important to use features that can well characterise a string pair by capturing the similarity between different variations while highlighting the difference between terms which are not synonymous. The considered features are character bigrams, prefixes and suffixes, numbers, acronyms, common and different tokens. An appropriate training set was prepared by processing sets of multiple names of the same metabolite in each of the databases. We found that the characteristic differences that occur between metabolite names are essentially of a very similar nature as those occurring between protein names, allowing the algorithm to perform efficiently. The result of this process consisted in a list of matched names with an associated of their identity.

### Chemical structure similarity

The formula of a metabolite theoretically confines the search for matching metabolites considerably. However it is not usable as a unique identifier. Two metabolites can have the same global formula and be completely different chemically because of the various possible structures of atoms. For example, 2-carboxy-D-arabinitol 1-phosphate, L-galactose-1-phosphate, D-hexose 6-phosphate and alpha-D-mannose 1-phosphate all have the same formula C_6_H_13_O_9_P. On the other hand, two metabolites that are identical may be represented by slightly different chemical formulae, either because an error is present in one source or because of chemical modifications (such as pH-dependent breakdowns of carboxylic groups).

All chemical structure processing was implemented in Pipeline Pilot workflows [24]. Input structures from KEGG and AraCyc were first converted to canonical SMILES, a unique line representation of two-dimensional molecular structure [25]. Exact structural matches occurred where SMILES strings were identical.

In previous metabolic network reconstructions it was observed that equivalent metabolites differed across sources in a number of ways. These included stereochemical differences (due to varying levels of detail about chiral centres or configurations about double bonds), tautomeric variants (where proton localisation differs), and charge states. To identify differences of these types SMILES strings were matched after purging stereochemical information from structure, or after calculation of the canonical tautomer, or following recalculation of ionisation at pH 7.4.

One further important way in which equivalent metabolites from different sources were noted to differ was by simple structural errors in their representation, such as incorrect bonding or missing functional groups. Providing that the majority of the structure is correctly specified then corrections for errors of this type can be suggested by standard cheminformatics molecular similarity measures [26] (here using the ECFP_4 molecular fingerprint and Tanimoto similarity metric).

Whereas exact matches of the original canonical SMILES strings provides an unambiguous mapping of metabolites across data sources, the other types of matches are only approximate (they may or may not be correct) and so require further manual checking. The approximate matching algorithms do, however, significantly reduce the manual checking workload.

### Complete process

**Figure 1:**
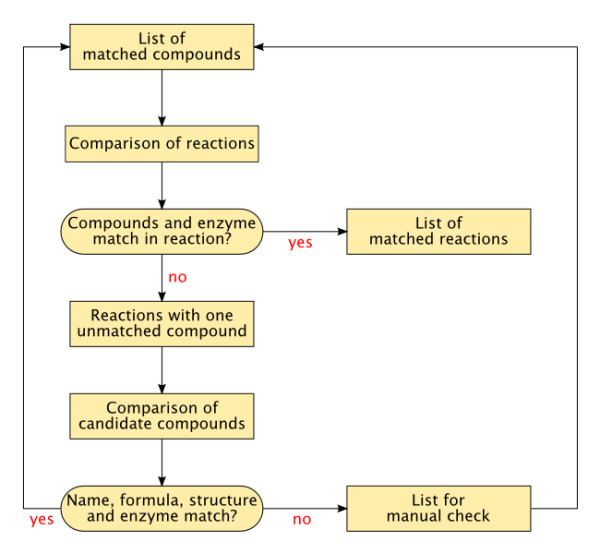
**Steps of the network reconstruction process**.

An iterative approach was adopted to integrate the different features of metabolite identification (Figure 1). The first step consisted in creating a list of mapped metabolites as a starting point. Those metabolites have been allocated by searching for Kegg references in AraCyc. For some of its compounds AraCyc provides the unique Kegg identifier, allowing the undoubted matching of a first set of compounds. The next step consisted in searching for all reactions in both databases that contain those compounds. More specifically, we considered those reactions where all compounds were already identified or only one was missing. Reactions where all compounds were identified could subsequently be compared, and if all compounds and the catalysing enzymes were the same, the reactions were accepted as being the same. In those reactions where one compound was missing, the known compounds were compared to each other. If two reactions in AraCyc and Kegg had the same number of metabolites and all known metabolites were the same, the respectively unknown compounds in each reaction were accepted as candidates for being identical. If the catalysing enzyme in both reactions was the same, and the name strings had a high probability of being similar, and the structures or formulae were equal, then two candidate compounds were accepted as being identical.

At the end of the first iteration step, new compounds were added to the list of matched compounds and a new iteration was started. The whole process was repeated several times until no additional matched compounds could be found. During the process, compounds that gave a positive result in some but not all of the before-mentioned features were copied into a separate list that was manually examined. The positive results of these checks were added to the list of compounds in order to improve the outcome of the next iteration.

The final lists of matched compounds and reactions were given new unique identifiers for the new reconstructions. These matched compounds and reactions constitute the first or *core (yellow) metabolic network*. Compounds and reactions that were not matched in both databases were assigned to the second or third network depending on an additional process. In the *intermediate (green) network*, we included reactions for which either the full set of substrates or the full set of products belonged to the core network. Such reactions are likely to be valid as they have a strong connection to the core network, but may be insufficiently or inconsistently described resulting in discrepancies between databases or their absence in one of them. Compounds involved in such reactions and not already included in the core model were also added to the intermediate network. All remaining compounds and reactions were included into the *complete (blue) network*.

A general problem in metabolic models is caused by the fact that many compounds have different protonation states depending on the pH. There is no pH consistency in formulae found in databases, and AraCyc does not represent protons in reactions. As a consequence, we neglected differences in proton content between formulae and did not represent protons in reactions either. A strict balance of hydrogen atoms thus cannot be expected in our reconstructions.

### Stoichiometric consistency validation

Incorrect definition of reaction stoichiometries often results in stoichiometric inconsistencies - a common type of modelling error, defined as contradictions between the fundamental physical constraints of mass positivity and mass conservation [27]. An example is shown below:

R1: A ↔ B

R2: A ↔ B + C

In this network, the metabolite C cannot be assigned any positive molecular mass without violating the mass balance in the whole system. Stoichiometric inconsistencies are often caused by violations of atomic balance, by ambiguous generic metabolite definitions (e.g. "primary alcohol") and by inclusion of polymers with variable polymerisation degrees and units (e.g. starch and protein).

Stoichiometric consistency validation involves inspection of the left null-space (the null-space of the transposed stoichiometry matrix) and may include the following optional steps: Firstly, the stoichiometric consistency of the full network is verified. If the network is inconsistent, the non-conserved metabolites [28] are detected (e.g. C in the above example). Further, for each non-conserved metabolite, the minimal inconsistent net stoichiometries involving it are calculated (these are defined as net stoichiometries with empty sets of substrate or products, e.g. by subtracting the reaction R1 from R2, we obtain Ø ↔ C). Finally, the elementary leakage modes (minimal linear combinations of reactions resulting in inconsistent net stoichiometries) can be detected, e.g. (-R1, R2) in the above example. The localisation of such minimal inconsistent subnetworks helps to detect input errors in the reactions.

### Construction of SBML version

An SBML version of the core network was constructed using libSBML [29]. The file is MIRIAM compliant [30] with all species and reactions annotated with ontological terms, allowing their unambiguous identification and interpretation by third party software tools. Metabolites have been annotated with ChEBI, Kegg, PubChem, 3DMET and Lipidbank terms, along with InChI strings [31], while all enzymes are provided with an annotation linking them to the appropriate gene in the TAIR database [32]. All annotations are associated to an appropriate systems biology ontology (SBO) term.

### Network visualisation and analysis

A network representation was constructed from the complete (blue) reconstruction for topological analysis and visualisation. In this representation, compounds are nodes and reactions are edges. All substrates of a given reaction were connected to all products of that reaction. Isolated compounds were not included in the network representation. The Cytoscape software was used for network visualisation [33], and the NetworkAnalyzer plugin for Cytoscape was used for topological analysis [34]. Network properties of the three reconstructions are summarised in Table 1. The meaning of the parameters shown in Table 1 is briefly explained hereafter [35].

**Table 1:** Properties of the three metabolic networks.

	Core (yellow) network	Intermediate (green) network	Complete (blue) network
Number of nodes	770	1207	2288
Number of edges	2255	3792	6547
Network density	0.008	0.005	0.002
Network heterogeneity	2.223	2.623	3.362
Number of self-loops	0	8	31
Clustering coefficient	0.215	0.233	0.189
Connected components	6	5	28
Network diameter	8	8	10
Network centralisation	0.288	0.301	0.271
Average path length	3.114	3.158	3.286
Average connectivity	5.857	6.270	5.696

The meaning of the parameters given in this table is explained in the Methods section.

The number of *connected components *indicates how many disjoint subnetworks the network is broken into. A *self-loop *is a node connected to itself. The number of *shared neighbours *between two nodes is the number of nodes that are neighbours of both of them. The *shortest path length*, also called the *distance *between two nodes is the smallest number of edges that have to be crossed to go from one edge to the other. The *characteristic path length *is the average distance and the *network diameter *is the largest distance between two nodes in the network.

The *connectivity *of a node is the number of edges connected to it. The *network density *is a measure of how densely the network is populated with edges. A network that contains only isolated nodes has a density of 0, whereas a clique has a density of 1. The *network centralisation *is a measure of how strongly a network is focused around central nodes. Networks resembling a star have a centralisation close to 1, whereas decentralised networks have a centralisation close to 0. The *network heterogeneity *measures the variance of the connectivity and reflects the tendency of a network to contain hub nodes. The *clustering coefficient *of a given node *n *is a ratio between the number of edges between the neighbours of *n *and the maximum number of edges that could possibly exist between them.

The *betweenness centrality C_b_*(*n*) of a node *n *is computed as follows:

$$C_{b}(n)=\frac{2}{\left(N-1)(N-2\right)}\sum_{s\neq n\neq t}\frac{\sigma_{st}(n)}{\sigma_{st}},$$

where *s *and *t *are nodes in the network different from *n*, *σ_st _*is the number of shortest paths from *s *to *t*, *σ_st_*(*n*) is the number of shortest paths from *s *to *t *that *n *lies on, and *N *is the total number of nodes in the connected component that *n *belongs to. The betweenness centrality of each node is a number between 0 and 1. It reflects the amount of control that a node exerts over the interactions between other nodes in the network. A node acting as a bridge between different communities has a high betweenness centrality, while a node that lies inside a community has a low one.

The *closeness centrality C_c_*(*n*) measures how close a node *n *is to others in the same connected component. It is defined as follows:

$$C_{c}(n)=\frac{N-1}{\sum_{m\neq n}L(m,n)},$$

where *L*(*n*, *m*) is the length of the shortest path between *n *and *m*, and *N *is the total number of nodes in the connected component that *n *belongs to. The closeness centrality is a measure of how fast information can spread from a given node to other reachable nodes in the network.

## Results

### Metabolic reconstructions of *A. thaliana*

**Figure 2:**
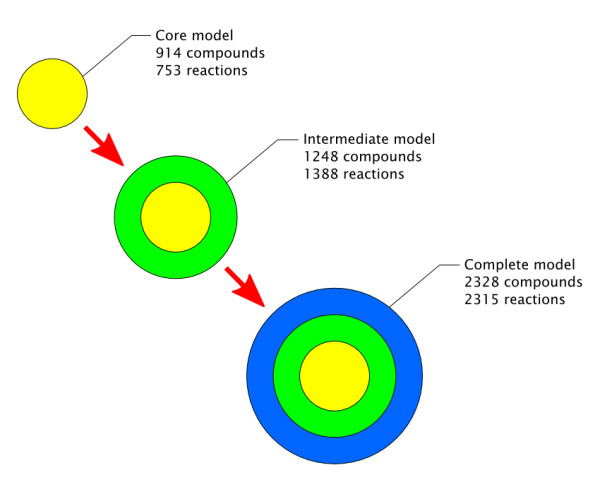
**Presentation and size of the three metabolic networks**.

Another important and often ignored aspect of metabolic network reconstruction is that different sources of data have different levels of certainty. A reaction consistently described by several independent sources should have a higher degree of reliability than a reaction described by a unique source. For this reason, we here present three different networks of *A. thaliana *metabolism corresponding to decreasing levels of confidence (Figure 2). The *core (yellow) network *contains only compounds and reactions which have been reliably identified in both databases and whose description is identical in both of them. The *intermediate (green) network *contains compounds and reactions found in only one database, but with a strong connection to the core network so that the absence of a perfect match is likely to be due to minor inaccuracies. The *complete (blue) network *contains all remaining compounds and reactions.

We present three metabolic networks of *A. thaliana *corresponding to different levels of confidence. The core (yellow) metabolic network only contains compounds and reactions that have been unambiguously identified and matched in the two databases (Additional file 1). These metabolites and reactions are expected to have been well characterised and experimentally observed, and are more likely to play an important role in the plant. This model is expected to cover most of the core metabolism of *Arabidopsis thaliana *(Table 1).

The intermediate (green) network additionally contains reactions from both databases where either the full set of substrates or the full set of products belongs to the core network (Additional file 2). Compounds involved in these reactions and not already included in the core network were also added to the intermediate network. Such reactions and compounds have a strong connection to the core network, but their confidence status is lower since they have not been unambiguously matched between both databases. The fact that a compound or reaction was found in only one database may be due to several factors: (i) our reconstruction algorithm may have failed to find the corresponding compound or reaction in the second database; (ii) a metabolite may be represented by a generic class in one database but by a specific compound in the other; (iii) one database may contain an inaccuracy, so that a metabolite or a cofactor is missing or incorrect in a reaction; (iv) a reaction or compound may indeed be absent from one database. Some of these causes might lead to the occurrence of double entries in the intermediate network.

All remaining reactions and compounds were added to the complete (blue) metabolic network, so that a comprehensive data set of all metabolic reactions and compounds described in *A. thaliana *and reactions contained is presented (Additional file 3). This reconstruction should be considered as a development set whose validity needs to be confirmed from additional sources. This file additionally contains a list of genes associated through each reaction in both databases.

**Figure 3:**
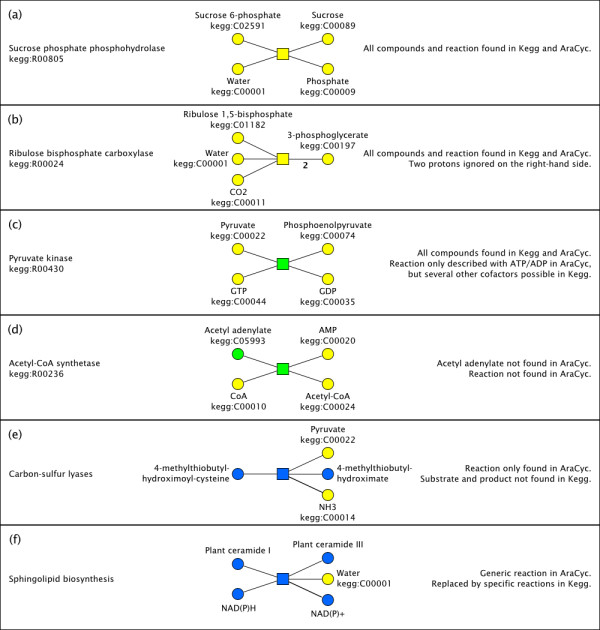
**Examples of reactions and their attribution to different confidence levels**. (a) Sucrose phosphate phosphohydrolase is identically described in both databases. (b) Ribulose bisphosphate carboxylase has a discrepancy in hydrogen content but protons involvement is ignored. (c) All substrates and product are validated in both databases, but the pyruvate kinase reaction with GTP/GDP as cofactors is only described in one database. (d) Acetyl adenylate and the acetyl-CoA synthetase reaction are only found in one database. (e) Both substrate and product are only found in one database. (f) Both sides of the reaction involve generic compound classes which are only used by one database.

For a better understanding of the different cases that result in the attribution of compounds and reactions to different reconstruction levels, several examples are shown in Figure 3. The sucrose phosphate phosphohydrolase reaction (a) is entirely yellow because it is identically described in both databases and all compounds were successfully matched to each other. So is the ribulose bisphosphate carboxylase reaction (b), even though it is described with two protons on the right hand side in Kegg but without in AraCyc, because we decided to ignore protonation states. The pyruvate kinase reaction with GTP/GDP as cofactors (c) is green even though all its substrates and products are yellow, because only Kegg describes the possibility of GTP/GDP involvement. Another pyruvate kinase reaction involving ATP/ADP is described in both databases and is included in the core (yellow) network. The acetyl-CoA synthetase reaction and acetyl adenylate (d) were not found in AraCyc, they were included into the intermediate (green) network because both acetyl-CoA and AMP are unambiguously identified. The carbon-sulfur lyase reaction (e) is blue because neither substrate nor product is known by AraCyc. The sphingolipid biosynthesis reaction (f) is blue because it uses generic metabolite classes in AraCyc that cannot be matched to specific metabolites in Kegg.

### Detailed coverage of metabolic pathways

We investigated the distributions of enzymes belonging to the three metabolic networks among Kegg pathways (Additional file 4). In most of the carbohydrate metabolism pathways, the core (yellow) metabolic network covers between 70% and 80% of all enzymes attributed by Kegg to these pathways. This proportion generally rises above 85% in the intermediate (green) network. In nucleotide metabolism pathways, 87% of the enzymes are covered by the core network and 91% by the intermediate network. For amino acid metabolism and secondary metabolites biosynthesis, these values are most of the time between 50% and 70% in the core network and 75% in the intermediate network. Lipid metabolism has a lower coverage with 40% to 60% of enzymes being in the core network and around 70% in the intermediate network. It is not surprising that core metabolic pathways are generally better covered, as these pathways should have been the most intensively experimentally analysed and the most accurately described.

**Figure 4:**
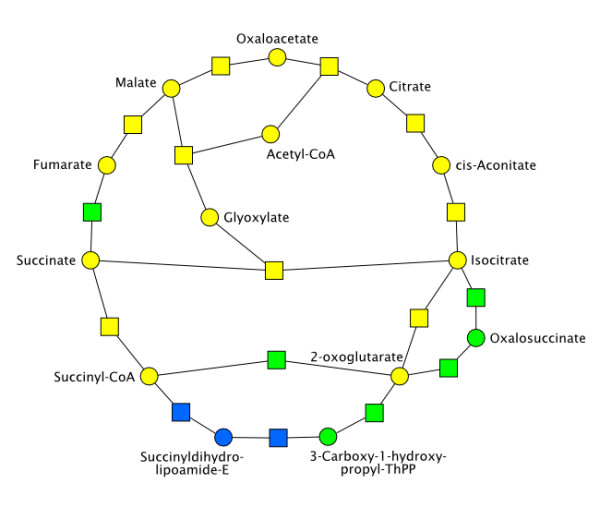
**Attribution of reactions and compounds in the tricarboxylic acid and glyoxylate cycles**. Most reactions and compounds in the tricarboxylic acid and glyoxylate cycles belong to the core (yellow) network. Succinate dehydrogenase, ketoglutarate dehydrogenase, and the transition between isocitrate and 2-oxoglutarate via oxalosuccinate belong to the intermediate (green) network. The transition between 2-oxoglutarate and succinyl-CoA via succinyldihydro-lipoamide-E belongs to the complete (blue) network.

As an illustration of the different levels of quality of our reconstructions, we describe the case of the tricarboxylic acid and glyoxylate cycles in detail (Figure 4). Most parts of the citrate cycle and the glyoxic shift have been reliably identified through the semi-automatic process and were included into the core (yellow) network. The remaining gaps are filled in the intermediate (green) network. The factors leading to some reactions and compounds not being included into the core network are detailed below:

(i) The succinate dehydrogenase reaction between succinate and fumarate was not included into the core network due to an ambiguity between various forms of ubiquinones and ubiquinols acting as cofactors. These compounds are represented by generic classes in Kegg, but by specific ubiquinone-8 and ubiquinol-8 compounds in AraCyc.

(ii) The transition between 2-oxoglutarate and succinyl-CoA is represented by a direct alpha-ketoglutarate dehydrogenase reaction in AraCyc. This reaction is not present in Kegg, which instead represents the transition by three different steps involving 3-carboxy-1-hydroxy-propyl-thiamine diphosphate and succinyldihydro-lipoamide-E.

(iii) Similarly, the transition between isocitrate and 2-oxoglutarate via oxalosuccinate is represented in Kegg but not in AraCyc. The direct isocitrate dehydrogenase reaction does appear in both databases.

### Stoichiometric consistency validation

The intermediate (green) and complete (blue) metabolic networks unsurprisingly contain many stoichiometric inconsistencies because these reconstructions contain generic metabolite classes (e.g. "alcohols"). The stoichiometric consistency validation of the core (yellow) network was successful with the exception of molecular hydrogen. This inconsistency inevitably follows from skipping protons from reaction definitions and currently cannot be resolved, given the inaccuracies in the input data. Achieving strict hydrogen and charge balancing would nevertheless be important to obtain a high quality genome-scale metabolic model, particularly for a photosynthetic organism [36].

### Network properties

**Figure 5:**
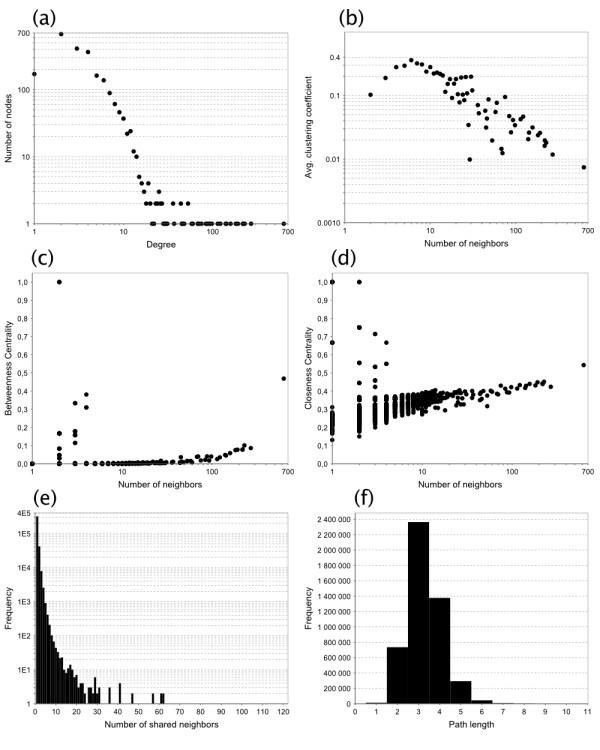
**Topological properties of the complete (blue) metabolic network**. (a) Node degree distribution. (b) Average clustering coefficient distribution. (c) Betweenness centrality. (d) Closeness centrality. (e) Shared neighbours distribution. (f) Shortest path length distribution. See Methods section for an explanation of network parameters.

We analysed the topological properties of the reconstructed metabolic networks in order to verify whether they were compatible with those of previously reported networks of other species (Table 1 and Figure 5). Different network representations can be used to represent systems of metabolic reactions, and the values of network parameters depend on the chosen representation. In this work metabolites were represented as nodes and reactions as edges. As the directionality of metabolic reactions is generally subject to ambiguity, edges were set to be undirected. Two metabolites were connected by an edge if they participate as substrate and product respectively in the same reaction. Common small molecules such as ATP, NADH, water, etc, were not removed from the network representation.

Distributions of the most important network properties are plotted in Figure 5 for the complete (blue) metabolic network. The intermediate (green) and core (yellow) metabolic networks exhibit similar distributions. Average network parameters are shown for the three networks in Table 1. The connectivity distribution of our metabolic reconstructions has the same allure, resembling a power-law, as universally observed in metabolic networks [37-39]. The average clustering coefficient is our reconstructions is close to 0.2, to be compared with reported values of 0.20 for *E. coli*, 0.23 for *S. cerevisiae*, and 0.28 for *H. pylori *based on the same network representation [39]. The average path length in our reconstructions is close to 3, which is the same as observed in many other metabolic networks based on the same network representation [37]. It is worth noticing that this value becomes significantly higher when common small molecules are removed from the network [40] or when an atomistic representation of metabolism is adopted [41].

In Arita's atomistic representation, the substrates and products of a reaction are connected only if carbon atoms can be traced between them. The Kegg RPAIR database provides atomic mappings between Kegg compounds, making it possible to construct an atomistic network for our core model. However, our intermediate and complete models contain some compounds that were not found in Kegg, therefore an atomistic network constructed from these models would be partially biased. For this reason, we provide a comparison between both network representations for the core model (Additional file 5), but the conclusions of this comparison can be extended to the entire network. In the atomistic network, there is a higher number of nodes of degree 1 and the degree distribution decreases more rapidly; the clustering coefficient and closeness centrality are lower, but the betweenness centrality is higher since nodes are less densely interconnected; the average path length is higher with its distribution peaking at 4, and the diameter of the network increases to 12 instead of 8 in the classical network.

**Figure 6:**
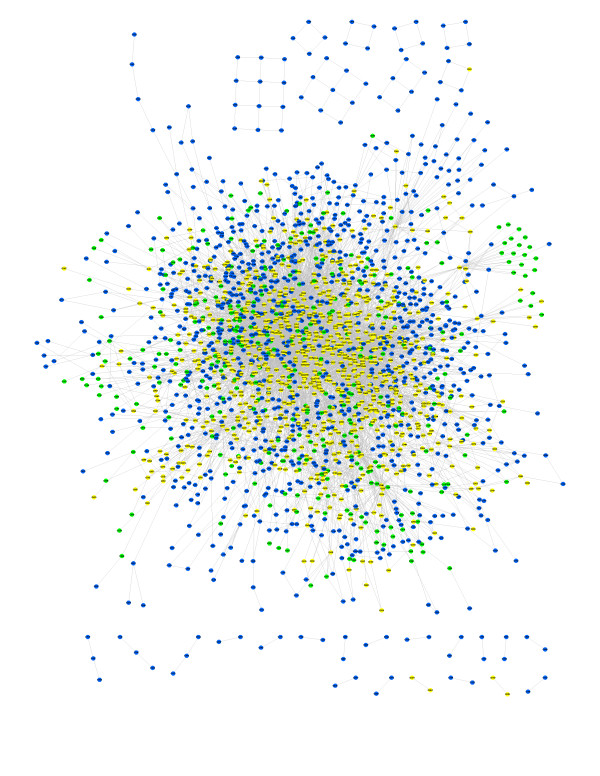
**Graphical representation of the complete network**. Nodes belonging to the core network are coloured in yellow, nodes added in the intermediate network are coloured in green, and nodes added in the complete network are coloured in blue.

The top ten hubs for the three metabolic networks are listed in Table 2. The connectivity of these hubs logically increases from the core to the complete networks, but their ranking is only marginally affected by the difference in confidence levels between networks. Water remains the most highly connected molecule in all cases, and nine out of ten molecules consistently appear the top ten ranking for all three networks. These hubs include most of the ubiquitous small molecules found in other metabolic networks, when they are not removed. A graphical network representation of the complete (blue) metabolic network is provided in Figure 6.

**Table 2:** Hubs of the three metabolic networks.

Core (yellow) network		Intermediate (green) network		Complete (blue) network	
**Metabolite**	**Degree**	**Metabolite**	**Degree**	**Metabolite**	**Degree**

Water	227	Water	369	Water	628
ATP	117	Oxygen	169	Oxygen	270
ADP	107	NADP	160	ATP	229
NADPH	89	NADPH	158	NADP	219
Orthophosphate	88	ATP	155	NADPH	218
NADP	84	ADP	128	Carbon dioxide	192
Carbon dioxide	81	Carbon dioxide	118	Diphosphate	182
Oxygen	79	Orthophosphate	102	ADP	159
Diphosphate	77	Diphosphate	101	NAD	143
NAD	60	NAD	89	NADH	140

### Comparison with other models

Because of the lack of usage of standardised formats, nomenclatures and annotations, it is very challenging to compare different genome-scale metabolic models with each other. A systematic comparison of our reconstruction with the two other *Arabidopsis *models, hereafter referred to as "Poolman" [16] and "AraGEM" [17], would therefore require a long and cumbersome manual mapping of metabolites and reaction.

We have nevertheless carried out such a manual comparison for the pathway shown in Figure 4, *i.e*. the tricarboxylic acid and glyoxylate cycles (Table 3). This comparison provides interesting information about the characteristics of each model. Most reactions were correctly included in our reconstruction, with the exception of protons which were deliberately ignored. The isocitrate dehydrogenase and oxalosuccinate decarboxylase reactions were included in our model but not in the two others, although there is evidence that corresponding genes were identified in *Arabidopsis *(AT1G54340, AT1G65930, AT5G14590). The Poolman model additionally ignored the reactions involving 3-carboxy-1-hydroxypropyl-ThPP and succinyldihydrolipoamide-E, but these were included in the AraGEM model. Other discrepancies can be observed in the use of cofactors: the succinate oxidoreductase is represented with ubiquinone/ubiquinol in our and Poolman's models, but with flavin adenine dinucleotide (FAD/FADH2) in AraGEM; the 2-oxoglutarate oxidoreductase reaction uses NAD/NADH in our and Poolman's models, but ferredoxin in AraGEM; the Poolman model ignores the use of NADP/NADPH by the isocitrate oxidoreductase and malate oxidoreductase reactions; the AraGEM model ignores the use of GTP/GDP by the succinate:CoA ligase reaction; the water molecule is missing in some reactions of the Poolman model. These results illustrate the difficulty to reach a consensus between different models and the amount of manual literature research that would be necessary to solve all discrepancies. Nevertheless, as seen from this example, the quality of our semi-automatic reconstruction compares favourably to manual reconstructions.

**Table 3:** Comparison of tricarboxylic acid and glyoxylate cycle reactions in *Arabidopsis *reconstructions.

Reaction	Radrich	Poolman	AraGEM
Succinate + Ubiquinone ↔ Fumarate + Ubiquinol	OK	OK	With FAD/FADH2
Fumarate + H2O ↔ Malate	OK	OK	OK
Malate + NAD ↔ Oxaloacetate + NADH + H Malate + NADP ↔ Oxaloacetate + NADPH + H	No H	Only with NAD/NADH	OK
Oxaloacetate + ADP + Orthophosphate + Acetyl-CoA ↔ Citrate + ATP + CoA	OK	OK	OK
Citrate ↔ cis-Aconitate + H2O	OK	No H2O	OK
cis-Aconitate + H2O ↔ Isocitrate	OK	No H2O	OK
Isocitrate ↔ Succinate + Glyoxylate	OK	OK	OK
Glyoxylate + H2O +Acetyl-CoA ↔ Malate + CoA	OK	No H2O	OK
Isocitrate + NAD ↔ 2-oxoglutarate + CO2 + NADH + H Isocitrate + NADP ↔ 2-oxoglutarate + CO2 + NADPH + H	No H	Only with NAD/NADH; 2 H	OK
Isocitrate + NADP ↔ Oxalosuccinate + NADPH + H	No H	NA	NA
Oxalosuccinate + NADP ↔ 2-oxoglutarate + CO2 + NADPH + H	No H	NA	NA
2-oxoglutarate + CoA + NAD ↔ Succinyl-CoA + CO2 + NADH	OK	OK	With ferredoxin
2-oxoglutarate + Thiamine-pyrophosphate ↔ 3-carboxy-1-hydroxypropyl-ThPP + Co2	OK	NA	OK
3-carboxy-1-hydroxypropyl-ThPP + Lipoamide-E ↔ Succinyldihydrolipoamide-E + Thiamine-pyrophosphate	OK	NA	OK
Succinyldihydrolipoamide-E + CoA ↔ Succinyl-CoA + Dihydropiloamide-E	OK	NA	OK
Succinyl-CoA + ADP + Orthophosphate ↔ Succinate + ATP + CoA Succinyl-CoA + GDP + Orthophosphate ↔ Succinate + GTP + CoA	OK	OK	Only with ADP/ATP

"Radrich" refers to this work, "Poolman" to the model by Poolman *et al. *[16] and "AraGEM" to the model by de Oliveira Dal'Molin *et al. *[17]. OK indicates that the reaction in the model is identical to the first column, NA that it is not included in the model; other differences are explicitly described.

Like the Poolman model, our reconstruction does not distinguish between cellular compartments, but the AraGEM model distinguishes between cytosol, mitochondria, plastid, vacuole and peroxisome. Since the aim of this work is to present a methodology for the integration of different databases, our reconstruction is not immediately intended for FBA simulations. There is a trade-off between the search for a consensus and the gap filling required for FBA.

## Discussion

The number of published genome-scale metabolic network reconstructions has grown rapidly in recent years. After the first reconstructions were published for *E. coli *and *S. cerevisiae *[42,43] the number of such reconstruction has been growing quickly in recent years, covering many microorganisms, animals and human. A comprehensive description of the motivations and applications of such reconstructions has been presented by Feist and Palsson [11]. These applications include network property analysis, metabolic engineering, biological discovery, phenotypic assessment, and evolutionary analysis.

Very few reconstructions of plant metabolic networks have been undertaken so far, and yet many of the applications mentioned before take even higher relevance in plants. Metabolic engineering is of particular significance in plants and offers promising perspectives to improving production yields, enhancing the nutritional value of crops, and generating valuable molecules for pharmacology and energy production. High-quality and comprehensive models of plant metabolism will be crucial to allow these applications to be developed. The metabolic networks of plants are of a higher complexity than those of most other living species; it is therefore both relevant and timely to start investing efforts in the construction of such models.

Although *Arabidopsis thaliana *has been widely used as a model plant, its metabolic network has not been studied in great details and at a large scale. There has been renewed interest in *A. thaliana *metabolism recently. More than 170 secondary metabolites from seven different classes have been identified in *A. thaliana *[44], whose putative functions cover the defence against pathogens and herbivores, UV protection, resistance to oxidative stress, auxin transport, etc. Glucosinolates are known for their benefits to human nutrition and were found to play a fundamental role in the defence response against microbial and fungal pathogens [45,46]. Biosynthesis pathways of tocochromanols, a group of lipid antioxidants that are essential in human nutrition, have raised promising interest [47]. *A. thaliana *was also used as a model plant to study polyamine metabolism, which plays an essential role in stress tolerance [48], and flavonoid production, which inhibit or stimulate cell proliferation in different human cancer cell lines [49].

However many issues presently hamper the use of genome-scale metabolic reconstructions by the wider research community and industry. These issues include:

(i) A limited usage of standardised formats, nomenclatures and annotations. Many metabolic reconstructions are published in spreadsheet format, using customs identifiers and nomenclature. This is a considerable obstacle to the transfer of these reconstructions to other applications and to comparisons between different networks, making it challenging to compare *Arabidopsis *models with each other.

(ii) Limited coordination between different reconstruction efforts. One such coordination has recently lead to the publication of a consensus metabolic reconstruction of *S. cerevisiae *[7] and a similar effort is currently under way for the human metabolic network [50].

(iii) The absence of update mechanisms enabling the integration of the latest scientific discoveries by the wider research community into existing models.

(iv) The absence of universal quality and validation standards.

Although this work does not claim to address all these issues, we introduced in this work a few principles seeking to propose avenues for solutions and to raise awareness about current limitations. First, we provide an annotated SBML version of the core (yellow) metabolic network of *A. thaliana *(Additional file 6) and non-annotated SBML versions of the two other networks (Additional files 7 and 8). SBML has become the *de facto *standard for systems biology models and allows them to be used by the widest range of tools. Standardised annotations following SBO specifications ensure that metabolites and enzymes can be easily identified and linked with existing databases. However, for such formats to be universally adopted by the biochemical research community, efficient and user-friendly tools will need to be developed allowing the easy input and conversion of models to a well annotated and standardised format.

While large international meetings have proven successful to confront and integrate different existing metabolic reconstructions, mechanisms allowing a convenient integration of models as they are developed would be more efficient. We showed that by confronting and integrating two independent sources, we were able to semi-automatically reconstruct a core metabolic network of *A. thaliana*, whose properties are comparable to existing manually reconstructed networks of other species. Such mechanisms could be generalised by the use of common repositories, following the model used for gene sequences or protein structures, allowing users to deposit new reconstructions and enhance existing ones through a seamless integration process.

Last, all applications using genome-scale metabolic reconstructions do not necessarily require the same level of data quality. For network analysis, a relatively straightforward or automatic reconstruction may be sufficient, while for metabolic engineering or experimental design a highly accurate and well-annotated model is generally necessary. It is therefore important to keep track of the sources and validation level of data used in reconstructions, so that users are able to select the data with the appropriate level of confidence for their application. As a first step towards such a process, we here provide three reconstructions with different levels of confidence. The core (yellow) network has the highest confidence level and was proven to be stoichiometrically consistent, but has some gaps. For applications such as Flux Balance Analysis, a more continuous model can be preferable even though some reactions might be of a lower confidence level. The intermediate (green) network attempts to suit such needs by filling gaps through the inclusion of partial information. The complete (blue) network eventually contains the largest amount of available information, but with the restriction that some reactions may be unconfirmed and the risk of duplications. These different levels of network reconstructions provide baselines upon which future improvements can be built by the community to ultimately obtain a high-quality genome-scale of *Arabidopsis *metabolism [51].

The software tools developed for this work are provided in Additional file 9 as Java source code, together with a protocol describing their function. The protocol used to map chemical structures is additionally available online from the myExperiment website [52].

## Conclusion

In this work, we presented a methodology allowing an efficient semi-automatic reconstruction of metabolic networks via the integration of different databases, and applied this methodology to the plant *Arabidopsis thaliana*. The integration of different data sources significantly enhances the quality of a reconstruction and leads to quality standards that are comparable to manual reconstructions. A long-term and coordinated international effort will be desirable to provide comprehensive and accurate genome-scale metabolic models of plants, and to provide the infrastructure allowing widespread diffusion, frequent update, unimpeded compatibility and convenience of use of such models by the widest research community and industry.

## Authors' contributions

KR developed the methodology and implemented algorithms for network reconstruction and database integration. YT, PD, AG, NS implemented algorithms for network reconstruction and validation. GB implemented gene-to-reaction associations. JMS designed and coordinated the study and analysed results. KR, PD, AG, NS, JMS wrote the manuscript. All authors read and approved the final manuscript.

## Supplementary Material

Additional file 1 **Compound and reaction data of the core (yellow) metabolic network**. The first sheet contains the list of compounds and the second sheet the list of reactions. Each compound is identified by a local identifier consisting of "Ath_C" followed by a four-digit number, its Kegg identifier and AraCyc name. Each reaction is identified by a local identifier consisting of "Ath_R" followed by a four-digit number, its Kegg identifier and AraCyc name. The stoichiometry column describes the reaction using local compound identifier. Substrates and products are separated by the equal ("=") sign. The stoichiometry is always explicitly written even when it is one. The enzyme column lists the enzymes catalysing each reaction by their EC number. Click here for file

Additional file 2 **Compound and reaction data of the intermediate (green) metabolic network**. The first sheet contains the list of compounds and the second sheet the list of reactions. Each compound is identified by a local identifier consisting of "Ath_C" followed by a four-digit number, its Kegg identifier and AraCyc name. Each reaction is identified by a local identifier consisting of "Ath_R" followed by a four-digit number, its Kegg identifier and AraCyc name. The stoichiometry column describes the reaction using local compound identifier. Substrates and products are separated by the equal ("=") sign. The stoichiometry is always explicitly written even when it is one. The enzyme column lists the enzymes catalysing each reaction by their EC number. Click here for file

Additional file 3 **Compound and reaction data of the complete (blue) metabolic network**. The first sheet contains the list of compounds and the second sheet the list of reactions. Each compound is identified by a local identifier consisting of "Ath_C" followed by a four-digit number, its Kegg identifier and AraCyc name. Each reaction is identified by a local identifier consisting of "Ath_R" followed by a four-digit number, its Kegg identifier and AraCyc name. The stoichiometry column describes the reaction using local compound identifier. Substrates and products are separated by the equal ("=") sign. The stoichiometry is always explicitly written even when it is one. The enzyme column lists the enzymes catalysing each reaction by their EC number. The gene columns list genes associated to each reaction based on EC numbers. Click here for file

Additional file 4 **Distribution of enzymes in the three metabolic networks for each Kegg pathway**. The first two columns give the Kegg identifier and name of each pathway. The yellow columns give the number of enzymes from this pathway attributed to the core metabolic network and its percentage in relation to the total number of enzymes contained in the pathway. The green columns give the number of enzymes attributed to the intermediate metabolic network and its percentage in relation to the total number of enzymes. The blue column gives the number of enzymes contained in the complete network, which is equal to the total number of enzymes contained in the pathway. Click here for file

Additional file 5 **Comparison between topological properties of a classical and atomistic representation for the core (yellow) metabolic network**. Red colour is used for the classical network, orange for the atomistic network. (a) Node degree distribution. (b) Average clustering coefficient distribution. (c) Betweenness centrality. (d) Closeness centrality. (e) Shared neighbours distribution. (f) Shortest path length distribution. See methods section for an explanation of network parameters. Click here for file

Additional file 6 **Annotated SBML file of the core (yellow) metabolic network**. Click here for file

Additional file 7 **SBML file of the intermediate (green) metabolic network**. Click here for file

Additional file 8 **SBML file of the complete (blue) metabolic network**. Click here for file

Additional file 9 **Software and protocol for semi-automatic reconstruction (Java source code)**. Click here for file
