# Supplementary figures and images for: Integration of metabolic databases for the reconstruction of genome-scale metabolic networks
Source: BMC Syst Biol. 2010 Aug 16;4:114. doi: 10.1186/1752-0509-4-114 (PMC2930596; doi:10.1186/1752-0509-4-114)

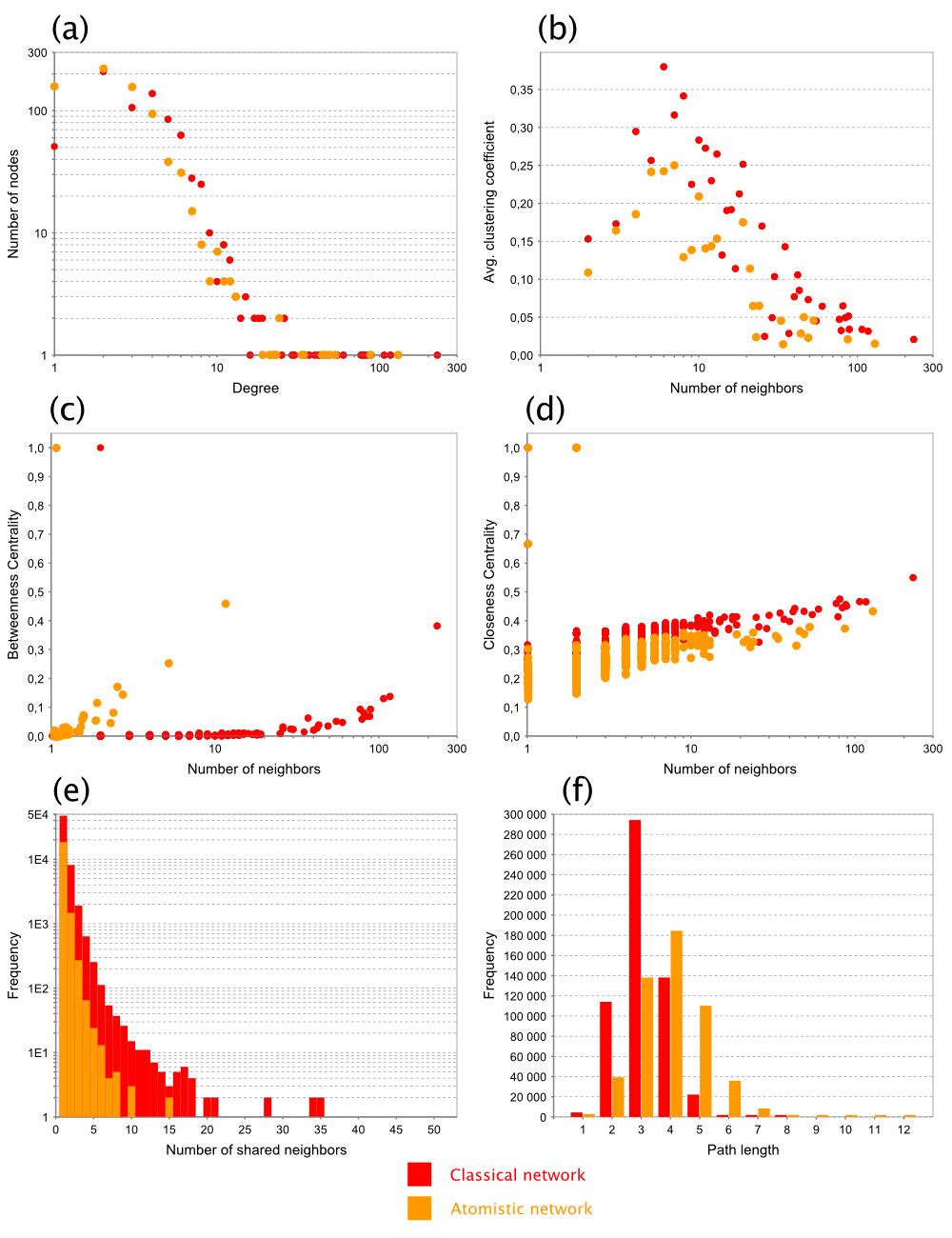

Supplement: Additional file 5 — Comparison between topological properties of a classical and atomistic representation for the core (yellow) metabolic network. Red colour is used for the classical network, orange for the atomistic network. (a) Node degree distribution. (b) Average clustering coefficient distribution. (c) Betweenness centrality. (d) Closeness centrality. (e) Shared neighbours distribution. (f) Shortest path length distribution. See methods section for an explanation of network parameters. [file 1752-0509-4-114-S5.PNG]
