# Supplementary material for: Integration of metabolic databases for the reconstruction of genome-scale metabolic networks
Source: BMC Syst Biol. 2010 Aug 16;4:114. doi: 10.1186/1752-0509-4-114 (PMC2930596; doi:10.1186/1752-0509-4-114)
Supplement: Additional file 9 — Software and protocol for semi-automatic reconstruction (Java source code). [file 1752-0509-4-114-S9.ZIP › readme.doc]

Program structure

The code is divided in two packages in order to allow the user a faster model building, once the basic steps have been performed. These two packages are called ‘AthModelPart1’ and ‘AthModelPart2’, and contain 18 and 12 classes respectively.

In part One the mapping of compounds and reactions is performed. This is a time consuming task. For this reason the second part has been conceived in order to easily make changes to the program and test them immediately, without having to wait for the operation of the program. Theoretically one package would be enough as in the second part many of the classes from part One are used as well, because they are providing some basic functions.

The results form part One are transferred to part Two. They are used there to create the three different model levels by allocating new IDs, writing stoichiometric matrices and network data for visualisation.

i) Package One – AthModelPart1

This package is conceived in such a way that, by running it once, all basic data for the final model generation is prepared. Thus this time consuming program does not have to be run twice and the modeller can concentrate on the real model generation in package Two. Basically, it reads in all data sources and extracts and compares the data, only outputting the information that is necessary for further proceeding.

In the following the classes and their general tasks are described, without detailing the variables and every step. Only necessary methods, input data and output data will be further explained and we advise the reader to read the code for a deeper insight. The classes contained in this model are (Figure A1):

- **AraCycData:** This class has the task of extracting useful information from the basic data format in the AraCyc database. Its methods thus build maps containing
  - All reactions mapped to the metabolites that are involved in them (method buildCpdRxnPairs).
  - A part of the information contained in ‘reactions.dat’, as specified in the parameter ‘type’, mapped to its reaction ID. A possible output would be all the data contained in the lines labelled by UNIQUE-ID, or all compounds that only occur at the left hand side of the reactions by asking for the information in lines labelled with LEFT. These labels have to be chosen as type parameters (method buildRxnData).
  - The formula set of a metabolite mapped to the compound ID of this metabolite (method buildFormulaSetAra).
- **AthKeggCpds:** This class extracts from the ath-folder all compounds and reaction IDs that are, according to KEGG, supposed to be in Arabidopsis thaliana and writes them in data sets.
- **CpdAssignment:** This class has the task to assign those compounds from AraCyc and KEGG which have the same formula to each other. There might be several matches in one database for only one compound in the other database. Thus multiple matches are written in a different map than unique matches, for this data to be processed in different ways.
- **CpdIdentification:** This class first searches for missing compounds that might get mapped, by comparing reactions in both databases where all but one compound has already been identified (method searchMissingCpd). If the chosen reactions are catalysed by the same enzyme, their compounds are compared by looking at their formulae or structure and the similarity of their names (findCorrespondence). If all features match, they will be added to the list of mapped metabolites. If at least one feature matches, they are added to the list for manual verification. Otherwise they will be discarded from being the same.
- **DoublesCount:** This class is for the verification of the uniqueness of the mapping of metabolites and does not influence the overall function of the program.
- **ImportExcel:** This class reads the metabolite structure data from its source file and converts it in a way that the format matches the formula of the other data.
- **KeggData:** This class has an analogous task as AraCycData for the management of KEGG data. Its methods are building maps containing:
  - The formula set of a metabolite mapped to the compound ID of this metabolite (method buildFormulaSetKegg).
  - A part, as specified in the parameter ‘type’, of the information contained in the map that is used as input parameter for this method, mapped to its reaction ID. A possible output would be all the data contained in the part labelled by ENZYMES, or NAME. These labels have to be chosen as type parameters and a column width has to be specified as well.
- **MainProgram:** The main program class manages the invocation of the different classes and thus the flow of information. We will give a more detailed description of its structure after having introduced all classes that it uses.
- **ManualCheck:** This class reads in the list of manually verified metabolites in order to add them to the mapped compounds.
- **MapOfCpds:** This class fulfils a basic function in the acquisition of the data from the initial databases of KEGG and AraCyc respectively. For both the data is written in maps to their respective unique IDs.
- **Printer:** This class provides methods to print out the final data into text documents and is not important for the general function of the program.
- **Reactions:** This method analyses the reaction data in more detail. The methods
  - map all compounds from AraCyc that occur in one reaction to the respective reaction ID (method writeAraCycReactions),
  - map all reactions from KEGG to the compounds they invoke, and reciprocally map all compounds to the reactions in which they occur as well (method writeKeggReactions),
  - replace the AraCyc IDs by KEGG IDs in all reactions for those metabolites that are already known. Thus KEGG and AraCyc reactions can be compared (methods replaceIDs).
- **RxnComparison:** This method compares reactions between AraCyc and KEGG. They are added to different categories, depending if they are definitely the same or if one or more compounds in them have not been identified.
- **SmallPrograms:** This class removes empty objects from an object list.
- **StringSimilarity:** This class reads the string similarity result data from ‘set1new_to_2new.txt’ and creates a map that only contains high probability matches.
- **StructureResults:** This class uses the structure comparison results to identify matching compounds.
- **SynonymMap:** This class changes the names from AraCyc and KEGG to a standard representation. Characters like ‘&’,’<’,’>’ and ‘;’ are removed.
- **Tables:** This class writes different data into one single object in order to add, for example, whole data groups into one list or set.

**Figure A1: Flowchart for AthModelPart1 (classes that do not influence the overall procedure like DoublesCount, Printer, Tables and SmallPrograms are not shown).**

The main program class invokes all the above classes in order to create three files that are used as input by AthModelPart2. The steps through this program are as follows:

- Creation of a set of all compounds that are in the Kegg ath-folder.
- Creation of maps with all compound data of Kegg and AraCyc respectively.
- Creation of maps with all Kegg and AraCyc compound names in standardised form.
- Creation of a map containing all string similarity results from the file "set1new_to_2new" where the probability of a mapping is bigger than 90%.
- Creation of a map of Kegg compound data for only those compounds that are in the Kegg ath-folder.
- Breakdown of Kegg ath data into mapped and unmapped compounds.
- Creation of a map containing all unmapped Kegg ath compounds and their formulae in a set representation.
- Creation of a map where all possible formula matches between AraCyc and Kegg are tracked.
- Writing of all mapped compounds into one map, which will be step by step completed in the course of the program.
- Creation of maps containing all reaction data from Kegg and AraCyc respectively.
- Preparation of classes and maps for comparisons in the iterative process.
- Creation of maps with enzyme data from Kegg and AraCyc respectively.
- Check for reactions in AraCyc that are duplicated because they are either listed twice in both directions or they are catalysed by more than one enzyme.
- Creation of a map with results on compound structures where already mapped compounds are excluded from the search.
- Start of the iteration process, which is repeated until no further compounds can be mapped to each other. During this process, all mapped compounds are first identified and their AraCyc IDs are replaced by the corresponding Kegg ID. Then the reactions are compared and compounds are identified that might be the same. Formulae and string similarity of those compounds are verified and matching compounds added to a map. Compounds where at least one of the features is common are put into another map to be manually verified.
- Check if any compound has wrongly been mapped to more than one other.
- Print files that are necessary for AthModelPart2.

The files for AthModelPart2 are:

- mAllRxnsMatch.txt: List of all reactions that have been identified in the format:
  AraCyc-reaction ID ‘empty space’ KEGG-reaction ID
- mappedCpds.txt: List of all metabolites that have been identified in the format:
  KEGG-compound ID ‘empty space’ AraCyc-compound ID
- mMinOneKeggCpd.txt: List of all AraCyc-reactions which contain at least one identified compound.

ii) Package Two – AthModelPart2

The second package only works with the mapped compounds and reactions. It takes the results of part One and verifies the stoichiometry before allocating new IDs and writing the stoichiometric matrix and the network data. Nine of its twelve classes have been taken from AthModelPart1 to filter the data in an appropriate way. The main task is accomplished by only three new classes:

- **IDs:** This class allocates new common IDs to the mapped compounds and reactions.
- **Stoichiometry:** This class takes all mapped reactions and checks their stoichiometry for identity.
- **MainProgram:** The main program invokes all the above classes in order to print the results of the three model levels into text documents. After preparing the data by using the classes from the first part, it is assured that all reactions are also stoichiometrically identical. Then, new IDs are allocated. In this class the modeller can choose which model level he wants to create by commenting and uncommenting parts of the code. The parts that have to be in- or excluded are clearly denoted in the program.
  If model two is built, ‘mMinOneKeggCpd.txt’ has to be read, in order to know which reactions have to be added to the data of the first model to build the second one.
  If model three is built, all remaining data is added.

iii) Output file structure

In this section, the content of all files that are printed out throughout the running of the two parts and their data format are explained.

**Part1**

- mAllRxnsMatch.txt: List of all reactions that have been identified in the format:
  AraCyc reaction ID ‘empty space’ KEGG reaction ID
- mappedCpds.txt: List of all metabolites that have been identified in the format:
  KEGG-compound ID ‘empty space’ AraCyc-compound ID
- mMinOneKeggCpd.txt: List of all AraCyc-reactions which contain at least one identified compound.

Besides the three main files, other files are written in the second part to keep track of the data contained in KEGG-ath. In this folder, which organises the Arabidopsis thaliana data in pathway subdivisions, some pathways do not contain compounds or reactions, or do contain glycans which are excluded from our approach. In order to let future modellers know which compounds were included in the final KEGG-Ath data and which pathways were discarded, the following text documents have been printed:

- KeggAthCpdList.txt: List of compounds that are supposed to be in Arabidopsis thaliana according to KEGG. Each line contains one compound-ID string.
- KeggAthGlyList.txt: List of glycans that are supposed to be in Arabidopsis thaliana according to KEGG. Each line contains one glycan-ID string.
- KeggAthReactions.txt: List of reactions that are supposed to be in Arabidopsis thaliana according to KEGG. Each line contains one reaction-ID string.
- KeggPWdiffConf.txt: List of all pathways that do not contain compounds. Each line contains the name of one pathway.
- KeggPWdiffRn.txt: List of all pathways that do not contain reactions. Each line contains the name of one pathway.

**Part2**

- mNewRxnID1.txt: List of new reaction IDs of model one and their correspondences in KEGG and AraCyc.
  Format: New ID ‘tab’ KEGG ID ‘tab’ AraCyc ID
- mNewCpdID1.txt: List of new compound IDs of model one and their correspondences in KEGG and AraCyc.
  Format: New ID ‘tab’ KEGG ID ‘tab’ AraCyc ID
- mNewCpdIDNew.txt: List of new compound IDs of model two or three, depending on which one has been selected, and their correspondences in KEGG and AraCyc.
  Format: New ID ‘tab’ KEGG ID ‘tab’ AraCyc ID
- mNewRxnIDNew.txt: List of new reaction IDs of model two or three, depending on which one has been selected, and their correspondences in KEGG and AraCyc.
  Format: New ID ‘tab’ KEGG-ID ‘tab’ AraCyc-ID
- NetworkData.txt: Data for network visualisation in Cytoscape. It contains a list in which every line has two compounds that are linked to each other in the network.
  Example: the reaction ‘metabolite1 + metabolite2 ⇔ metabolite3 + metabolite4’ will be written in this file as follows:
  Metabolite1 ‘empty space’ metabolite3
  Metabolite1 ‘empty space’ matabolite4
  Metabolite2 ‘empty space’ metabolite3
  Metabolite2 ‘empty space’ matabolite4
- mStoMatrix.txt: Stoichiometric matrix of the current model type (1, 2 or 3) that has been built. All reactions containing stoichiometric coefficients that are not numbers but generics, like ‘m’ or ‘n’, have been excluded in the version on which the consistency check was performed, because such reactions are not supported by the algorithm.
  Format: every line contains the data for one reaction.
  Example: in a system with 10 metabolites, a reaction of the type ‘2 metabolite1 + metabolite4 ⇔ metabolite6 + 3 metabolite10’ is represented as follows:
  -2 0 0 -1 0 1 0 0 0 3, with empty spaces between each coefficient.
- Reactions.txt: List of new reaction IDs of the current model type and their correspondences in KEGG and AraCyc. The metabolites and coefficients are added in this document.
  Format: New ID ‘tab’ KEGG-ID ‘tab’ AraCyc-ID ‘tab’ ‘c1 m1 + c2 m2 ⇔ c3 m3 + c4 m4’
  where ‘c1’ means coefficient of ‘m1’ which is metabolite1.
- Reactions+Enzymes.txt: Same data as in Reactions.txt, but for each reaction the catalysing enzymes have been added at the end of each reaction line.
